# Fluid management for sepsis-induced hypotension in patients with advanced chronic kidney disease: a secondary analysis of the CLOVERS trial

**DOI:** 10.1186/s13054-024-05019-6

**Published:** 2024-07-11

**Authors:** Anselm Jorda, Ivor S. Douglas, Thomas Staudinger, Gottfried Heinz, Felix Bergmann, Rainer Oberbauer, Gürkan Sengölge, Markus Zeitlinger, Bernd Jilma, Nathan I. Shapiro, Georg Gelbenegger

**Affiliations:** 1 Department of Clinical Pharmacology, Medical University of Vienna, Waehringer Guertel 18-20, 1090 Vienna, Austria; 2 Department of Medicine, Pulmonary Sciences and Critical Care, Anschutz Medical Campus, Denver Health and University of Colorado, Denver, CO USA; 3 Department of Medicine I, Medical University of Vienna, Vienna, Austria; 4 Division of Cardiology, Department of Medicine II, Medical University of Vienna, Vienna, Austria; 5 Division of Nephrology and Dialysis, Department of Medicine III, Medical University of Vienna, Vienna, Austria; 6 Department of Emergency Medicine, Beth Israel Deaconess Medical Center, Harvard Medical School, Boston, MA USA

**Keywords:** Bacteremia, Septic shock, Hypervolemia, Vasopressor, Chronic kidney disease, Dialysis

## Abstract

**Background:**

Early fluid management in patients with advanced chronic kidney disease (CKD) and sepsis-induced hypotension is challenging with limited evidence to support treatment recommendations. We aimed to compare an early restrictive versus liberal fluid management for sepsis-induced hypotension in patients with advanced CKD.

**Methods:**

This *post-hoc* analysis included patients with advanced CKD (eGFR of less than 30 mL/min/1.73 m^2^ or history of end-stage renal disease on chronic dialysis) from the crystalloid liberal or vasopressor early resuscitation in sepsis (CLOVERS) trial. The primary endpoint was death from any cause before discharge home by day 90.

**Results:**

Of 1563 participants enrolled in the CLOVERS trial, 196 participants had advanced CKD (45% on chronic dialysis), with 92 participants randomly assigned to the restrictive treatment group and 104 assigned to the liberal fluid group. Death from any cause before discharge home by day 90 occurred significantly less often in the restrictive fluid group compared with the liberal fluid group (20 [21.7%] vs. 41 [39.4%], HR 0.5, 95% CI 0.29–0.85). Participants in the restrictive fluid group had more vasopressor-free days (19.7 ± 10.4 days vs. 15.4 ± 12.6 days; mean difference 4.3 days, 95% CI, 1.0–7.5) and ventilator-free days by day 28 (21.0 ± 11.8 vs. 16.5 ± 13.6 days; mean difference 4.5 days, 95% CI, 0.9–8.1).

**Conclusions:**

In patients with advanced CKD and sepsis-induced hypotension, an early restrictive fluid strategy, prioritizing vasopressor use, was associated with a lower risk of death from any cause before discharge home by day 90 as compared with an early liberal fluid strategy.

**Trial Registration:**

NCT03434028 (2018-02-09), BioLINCC 14149.

**Supplementary Information:**

The online version contains supplementary material available at 10.1186/s13054-024-05019-6.

## Background

Sepsis represents a major health care problem worldwide and is associated with a high treatment burden and mortality, contributing to an estimated 20% of all global deaths [1–3]. Intravenous fluid resuscitation is a cornerstone in the treatment of sepsis-induced hypoperfusion, aiming to increase preload, cardiac output, and oxygen delivery to tissues [4, 5]. In contrast, excess fluid replenishment may lead to volume overload which bears the risk of prolonged ventilation and increased mortality [6]. Current 2021 guidelines from the Surviving Sepsis Campaign (SSC) recommend the administration of 30 mL/kg body weight of intravenous crystalloid fluid within the first three hours of resuscitation with further fluid management being guided by dynamic parameters, such as stroke volume variation, pulse pressure variation, or echocardiography [7]. Fluid management in patients with advanced chronic kidney disease (CKD) who suffer from sepsis proves particularly challenging because these patients are prone to volume overload due to a dysregulated fluid balance and perturbed circulatory response to vasopressors. Moreover, advanced CKD itself is associated with an increased risk of death in patients with sepsis [8, 9]. Recommendations for fluid management in sepsis are largely based on low-quality evidence [10], and quality data for the subpopulation of patients with advanced CKD are mostly lacking. While retrospective data hint towards an acceptable fluid tolerance in patients with advanced CKD and sepsis during the fluid resuscitation phase [11, 12], in-depth prospective data, particularly involving the optimization and stabilization phases of fluid therapy in sepsis, are still missing.

The CLOVERS (Crystalloid Liberal or Vasopressor Early Resuscitation in Sepsis) trial randomized participants with sepsis-induced hypotension to an early restrictive treatment strategy (prioritizing vasopressors and lower intravenous fluid volumes) or liberal fluid treatment strategy (prioritizing higher volumes of intravenous fluids before vasopressor use) and was unable to detect a significant difference in mortality before discharge home by day 90 between the two treatment groups [13]. We hypothesized that patients with advanced CKD (defined as patients with estimated glomerular filtration rate [eGFR] of less than 30 mL/min/1.73 m^2^ or end-stage renal disease [ESRD] on chronic dialysis) and sepsis-induced hypotension might benefit from an early restrictive fluid strategy and aimed to test our hypothesis in a *post-hoc* analysis of the CLOVERS trial.

## Methods

### Study design

This *post-hoc* analysis of the randomized controlled CLOVERS trial aimed to investigate whether a restrictive fluid strategy, compared with a liberal fluid strategy, improves clinical outcomes in the subgroup of patients with advanced CKD. The design and results of the original CLOVERS trial have been published previously [13, 14]. A central institutional review board and NHLBI-appointed independent data and safety monitoring board reviewed and approved the original trial protocol. Data for this *post-hoc* analysis was obtained from the NHLBI Biologic Specimen and Data Repository Information Coordinating Center [15]. The Ethics Committee of the Medical University of Vienna waived the need for review of this study.

### Patient population

The CLOVERS trial enrolled adult participants (18 years or older) with a suspected or confirmed infection and hypotension caused by sepsis (systolic blood pressure below 100 mmHg despite an intravenous infusion of at least 1000 mL of crystalloid fluid). Key exclusion criteria were a period of more than 4 h since meeting the criteria for hypotension unresponsive to intravenous infusion of at least 1000 mL, a period of more than 24 h since admission to hospital, previous administration of at least 3000 mL of intravenous fluid during this episode, severe volume depletion due to causes other than sepsis, and the presence of fluid overload. Fluid overload included pulmonary or peripheral edema suggested by clinical signs (bilateral crackles) or radiologic findings (fluid overload on chest x-ray).

This *post-hoc* analysis included patients with advanced CKD, defined by an eGFR of less than 30 mL/min/1.73 m^2^ or a history of ESRD on chronic dialysis. Patients were included in this analysis if they met at least one of the following criteria: (i) the patient was on chronic dialysis at time of randomization, as documented in the case report form, (ii) an eGFR of less than 30 mL/min/1.73 m^2^ based on a (not acutely elevated) serum creatinine value from the previous year before randomization, and (iii) the baseline comorbidity assessment which was used to calculate the Charlson comorbidity index. We calculated eGFR values using the baseline serum creatinine, age, sex, and skin color with the Chronic Kidney Disease Epidemiology Collaboration 2021 (CKD-EPI) formula [16]. The criteria for advanced CKD in the Charlson Comorbidity Index used in the CLOVERS trials included either a serum creatinine of more than 3 mg/dL, a chart diagnosis of CKD stage 5 (eGFR of less than 15 mL/min/1.73 m^2^) or the need for dialysis.

### Trial procedures

The CLOVERS trial randomly assigned participants in a 1:1 ratio to either a restrictive fluid strategy (with early vasopressor use) or a liberal fluid strategy. The assigned fluid management protocol was followed for 24 h after randomization. The restrictive fluid protocol prioritized vasopressors as the primary treatment for sepsis-induced hypotension, with “rescue fluids” permitted in case of severe intravascular volume depletion. Norepinephrine was suggested as the primary vasopressor. The liberal fluid protocol recommended an initial 1000 mL intravenous bolus infusion of isotonic crystalloids. After clinical reassessment, another 1000 mL of isotonic crystalloid fluid were given in case of persistent volume depletion. Further fluid boluses were based on clinical triggers, such as tachycardia. The conduct of the study was supported by a trial team to improve protocol adherence. After the first 24 h, fluid management was no longer specified.

### Outcomes

The primary outcome was death from any cause before discharge home by day 90. Secondary outcomes included 28 day measures of the number of days free from ventilator use, days free from vasopressor use, days out of the ICU, and days out of the hospital. We also assessed the new onset of acute respiratory distress syndrome by day 7 and new intubation by day 28. The days free from renal-replacement therapy were only analyzed in the subset of patients with advanced CKD not previously receiving dialysis. Incidence of acute kidney injury (AKI) was compared between the two groups. AKI definitions followed those of the KDIGO (The Kidney Disease: Improving Global Outcomes) recommendations [17]: AKI stage I was defined as a short-term increase in serum creatinine by at least 0.3 mg/dL or an at least 1.5-fold increase in creatinine levels. AKI stage II and III were defined as a 2- to threefold increase and more than threefold increase in serum creatinine levels, respectively. Calculations were based on the serum creatinine value at randomization and the highest creatinine value within 6 days after randomization.

### Statistical analysis

Categorical variables are presented using numbers with percentage (%). Continuous variables are summarized using mean with standard deviations (SD) or medians with interquartile ranges (IQR), depending on the data distribution. Baseline differences between the restrictive fluid group and liberal fluid group were tested using Fisher's exact test or independent *t* test (or Wilcoxon rank-sum test in case normal distribution cannot be assumed). Analysis of the primary outcome (death before discharge home by day 90) used Kaplan–Meier time to event analysis. *P* values of the time-to-event analyses were calculated using the log-rank test. Hazard ratios of death were calculated using a Cox proportional-hazards model. Visual inspection of the Schoenfeld residuals revealed no violation of proportional hazards assumptions. For all other outcomes, we report mean differences with 95% confidence intervals. For the primary outcome, we used forest plots to assess treatment heterogeneity between patients with advanced CKD not receiving dialysis, in patients with advanced CKD receiving dialysis, and patients without established advanced CKD (i.e., all remaining participants who did not meet the criteria for advanced CKD). We performed univariate Cox regression analyses to identify variables associated with death before discharge home by day 90. Variables with a *p* value below 0.1 were selected for a multivariate Cox regression model to adjust the primary outcome comparison for residual confounding. To determine the risk of collinearity, univariate correlation coefficients between the independent variables and the variance inflation factor (VIF) were computed. Univariate correlation coefficients below 0.4 were considered weak and therefore acceptable for the multivariate model. The VIF is a measure of how much the variance of an estimated regression coefficient is increased due to collinearity. In this study, VIF values > 2.5 were interpreted as meaningful collinearity and values > 10 as significant collinearity. All analyses used an intention-to-treat approach. All *P* values are two-sided. Due to the exploratory nature of this *post-hoc* analysis, no adjustment of *P* values or confidence intervals for multiple comparisons was made. Therefore, the results of this analysis can only be hypothesis-generating and should not be interpreted as an inference for treatment decisions. Analyses and data visualization were conducted in R statistical software, R version 4.1.2 (2021-11-01) and RStudio Version 2023.09.1 + 494.

## Results

### Participants and baseline characteristics

**Fig. 1:**
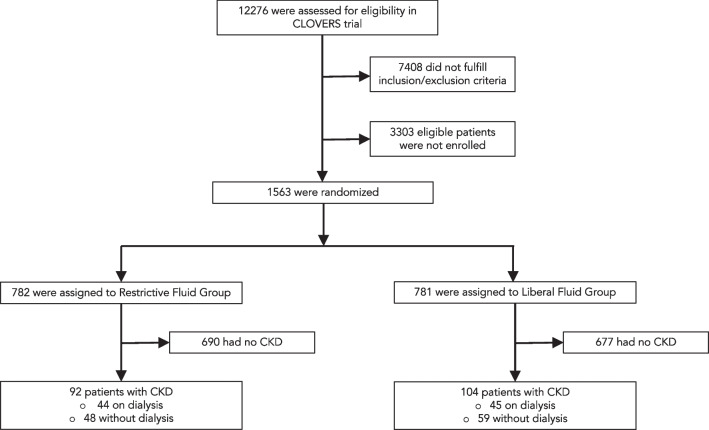
Flow chart of the study

From March 2018 to January 2022, the CLOVERS trial assessed 12,276 patients from 60 U.S. study centers for eligibility. Of these, 1563 patients were included. A total of 782 participants were assigned to the restrictive fluid group and 781 to the liberal fluid group. The trial was prematurely terminated following the recommendation of the data and safety monitoring board which concluded futility due to a lack of between-group differences in the outcomes. After excluding 1367 participants without advanced CKD, this *post-hoc* analysis comprised 92 participants with advanced CKD (eGFR of less than 30 mL/min/1.73 m^2^ or history of ESRD on chronic dialysis) in the restrictive fluid group and 104 participants with advanced CKD in the liberal fluid group. The study flow chart is provided in Fig. 1.

Within the advanced CKD subgroup, baseline characteristics were similar between the restrictive fluid group and the liberal fluid group (Table 1). Forty-four (47.8%) of 92 participants in the restrictive fluid group and 45 (43.3%) of 104 participants in the liberal fluid group received chronic dialysis. Participants in the restrictive fluid group and the liberal fluid group had received similar amounts of intravenous fluid before randomization (mean ± SD, 1844 ± 672 and 1854 ± 627 ml, respectively). Vasopressors were used at randomization in 23 (25.0%) of 92 participants in the restrictive fluid group and 27 (26.0%) of 104 participants in the liberal fluid group.

**Table 1:** Characteristics of the participants at baseline

	Overall	Restrictive fluid group	Liberal fluid group	*p*
Number of participants	196	92	104	
Age, years (median (IQR])	65 (54 to 74)	62 (52 to 73)	67 (57 to 74)	0.064
BMI, kg/m^2^ (median [IQR])	27.5 (23.4 to 36.3)	29.3 (24.0 to 40.4)	26.5 (22.8 to 31.7)	0.028
Male sex (n [%])	108 (55.1)	48 (52.2)	60 (57.7)	0.528
Ethnicity (n [%])				0.401
Hispanic or Latino	35 (17.9)	18 (19.6)	17 (16.3)	
Not Hispanic or Latino	150 (76.5)	67 (72.8)	83 (79.8)	
Not reported	11 ( 5.6)	7 ( 7.6)	4 ( 3.8)	
Race (n [%])				
Asian	8 (4.1)	2 (2.2)	6 (5.8)	0.364
White	110 (56.1)	56 (60.9)	54 (51.9)	0.265
African American	54 (27.6)	22 (23.9)	32 (30.8)	0.362
Not reported	23 (11.7)	11 (12.0)	12 (11.5)	1.000
COPD (n [%])	31 (15.8)	11 (12.0)	20 (19.2)	0.231
Heart failure (n [%])	42 (21.4)	22 (23.9)	20 (19.2)	0.533
Hypertension (n [%])	126 (64.3)	53 (57.6)	73 (70.2)	0.092
Coronary artery disease (n [%])	43 (21.9)	20 (21.7)	23 (22.1)	1.000
Neoplasia (n [%])				0.037
Not present	163 (83.2)	82 (89.1)	81 (77.9)	
Present	13 (6.6)	6 (6.5)	7 (6.7)	
Present with metastasis	20 (10.2)	4 (4.3)	16 (15.4)	
Diabetes (n [%])				0.985
Not present	101 (51.5)	47 (51.1)	54 (51.9)	
Present with end organ damage	50 (25.5)	24 (26.1)	26 (25.0)	
Uncomplicated	45 (23.0)	21 (22.8)	24 (23.1)	
Chronic dialysis (n [%])	89 (45.4)	44 (47.8)	45 (43.3)	0.620
Location of randomization (n [%])				0.287
Emergency department	172 (87.8)	84 (91.3)	88 (84.6)	
ICU	23 (11.7)	8 (8.7)	15 (14.4)	
Other	1 (0.5)	0 (0.0)	1 (1.0)	
SOFA score at randomization (median [IQR])	4 (2 to 6)	4 (2 to 5.25)	4 (2.75 to 7)	0.195
Volume administered before randomization (mean ± SD)	1850 ± 647	1844 ± 672	1854 ± 627	0.914
Vasopressor use at randomization (n [%])	50 (25.5)	23 (25.0)	27 (26.0)	1.000
ARDS at randomization (n [%])	4 (2.0)	3 (3.3)	1 (1.0)	0.529
Serum lactate at randomization, mmol/L (median [IQR))	2.6 (1.6 to 4.2)	2.8 (1.6 to 4.1)	2.5 (1.6 to 4.4)	0.817
Mechanically ventilated at randomization (n [%])	55 (28.1)	23 (25.0)	32 (30.8)	0.461

*ARDS* Acute respiratory distress syndrome, *COPD* chronic obstructive pulmonary disease, *SOFA* sequential organ-failure assessment

Table S1 shows the baseline characteristics of participants with advanced CKD not receiving dialysis (n = 107), participants with advanced CKD receiving dialysis (n = 89), and participants excluded from this *post-hoc* analysis because of absent advanced CKD (n = 1367), irrespective of their group assignment. Self-reported African American race was significantly more prevalent in the group of participants with advanced CKD not on dialysis (22 [20.6%]) and in the group of participants with advanced CKD on dialysis (32 [36.0]) than in the group of participants without advanced CKD (193 [14.1%]).

### Protocol-guided resuscitation treatments

Within the first 6 h after randomization, the median volume of intravenous fluid was 424 mL (IQR 50 to 950) in the restrictive fluid group and 2300 mL (IQR 2000 to 3000) in the liberal fluid group, resulting in a mean difference of −1776 mL (95% CI −2025 to −1526) (Table S2 and Fig. S1). Similarly, the total median volume of intravenous fluid during the first 24 h after randomization was lower in the restrictive fluid group (median 1200 mL, IQR 490 to 2373) than in the liberal fluid group (median 3325 mL, IQR 2500 to 4641), with a mean difference of −1667 mL (95% CI −2262 to −1072) (Table S2, Fig. S2).

The urinary output within 24 h (excluding patients who were anuric) was similar between the restrictive group (median 345 mL, IQR 0 to 989) and the liberal fluid group (median 390 mL, IQR 0 to 1000). The net positive fluid balance was significantly lower in the restrictive group (median 1087 mL, IQR 90 to 2427) was significantly lower than in the liberal fluid group (median 2961 mL, IQR 1900 to 3995), with a mean group difference of −1400 mL (95% CI −2133 to −667).

Vasopressors were more frequently administered in the restrictive fluid group (68 [73.9%] of 92) than the liberal fluid group (52 [50%] of 104), initiated earlier (mean difference, −2.4 h; 95% CI −4.3 to −0.5), and used for longer during the first 24 h after randomization (mean difference 6.4 h, 95% CI 3.6 to 9.2) (Fig. S3).

### Primary efficacy outcome

**Fig. 2:**
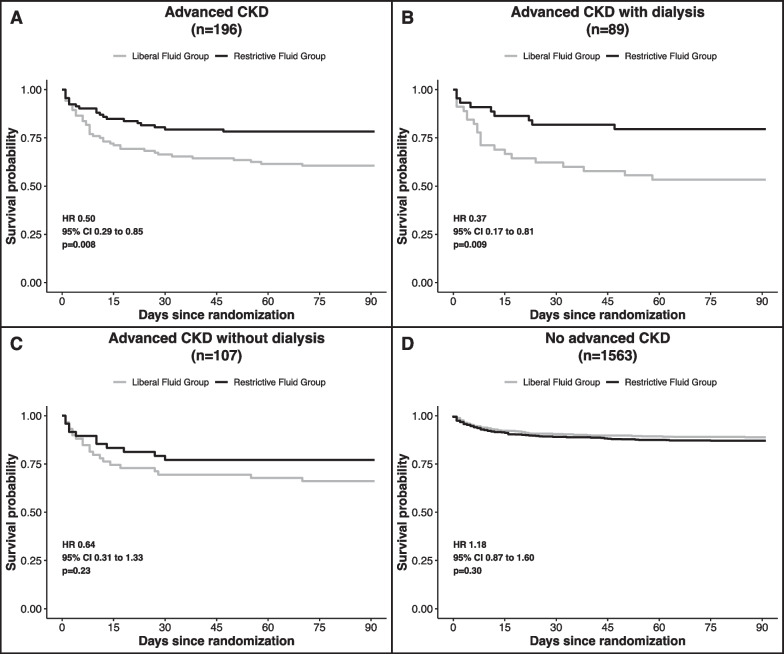
**A**–**D** Death from any cause before discharge home by day 90 (primary outcome) between the restrictive fluid group and liberal fluid group **A** in all patients with advanced chronic kidney disease (CKD), **B** in patients with advanced CKD receiving dialysis, **C** in patients with advanced CKD not receiving dialysis, (D) and in patients without advanced CKD

**Fig. 3:**
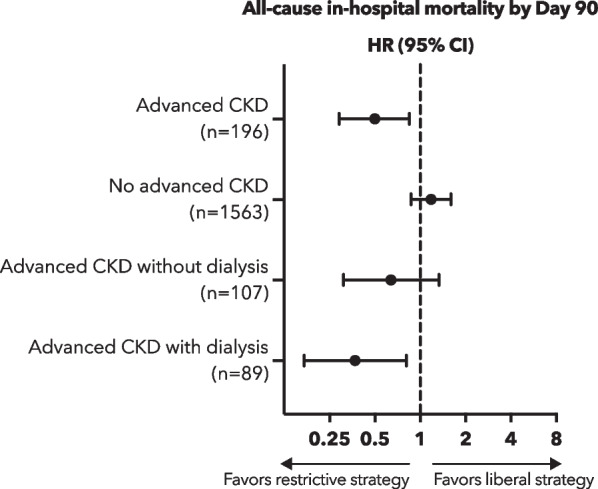
Effect estimates of the primary outcome (death from any cause before discharge home by day 90) between the restrictive fluid group and liberal fluid group in (i) patients with advanced chronic kidney disease (CKD), (ii) patients with no advanced CKD not receiving dialysis, (iii) patients with CKD receiving no dialysis, and (iv) patients with advanced CKD receiving dialysis

Death before discharge home by day 90 occurred in 20 participants (21.7%) in the restrictive fluid group and in 41 participants (39.4%) in the liberal fluid group (HR 0.5, 95% CI 0.29 to 0.85, *p* = 0.009) (Table 2 and Fig. 2A). Figure 3 shows the primary outcome in participants with advanced CKD not receiving dialysis, in participants with advanced CKD receiving dialysis, and participants without established advanced CKD. In the subgroup analysis of participants with advanced CKD receiving dialysis, the primary endpoint occurred in 9 (20.5%) of 44 in the restrictive fluid group and 21 (46.7%) of 45 participants in the liberal fluid group (HR 0.37, 95% CI 0.17 to 0.81) (Fig. 2B). In the subgroup analysis of participants with advanced CKD not receiving dialysis, the primary endpoint occurred in 11 (22.9%) of 48 in the restrictive fluid group and 20 (33.9%) of 59 participants (HR 0.64, 95% CI 0.31 to 1.33) (Fig. 2C). In participants without advanced CKD, the primary endpoint occurred in 89 (12.9%) of 690 in the restrictive fluid group and 75 (11.1%) of 677 participants (HR 1.18, 95% CI 0.87 to 1.6).

**Table 2:** Overview of primary and secondary study outcomes

	Overall	Restrictive fluid group	Liberal fluid group	Effect estimates with 95% CI	*P* value
Number of participants	196	92	104		
Death before discharge home by day 90 (n [%])	61 (31.1)	20 (21.7)	41 (39.4)	0.50 (0.29 to 0.85)^1^	0.009
Days free from ventilator use at 28 days (mean ± SD)	18.6 ± 13.0	21.0 ± 11.8	16.5 ± 13.6	4.5 (0.9 to 8.1)^2^	0.015
Days free from vasopressor use at 28 days (mean ± SD)	17.42 (11.8)	19.7 ± 10.4	15.4 ± 12.6	4.3 (1.0 to 7.5)^2^	0.010
Days out of the ICU by day 28 (mean ± SD)	18.1 ± 11.4	19.1 ± 10.9	17.2 ± 11.8	1.9 (-1.3 to 5.1)^2^	0.241
Days out of the hospital by day 28 (mean ± SD)	11.7 ± 11.2	13.3 ± 11.0	10.3 ± 11.3	2.9 (-0.2 to 6.1)^2^	0.067
New intubation with invasive mechanical ventilation by 28 days (n [%])	30 (17.4)	12 (14.8)	18 (19.8)	0.70 (0.3 to 1.7)^3^	0.427
ARDS onset between day 1 and day 7 (n [%])	6 (3.1)	1 (1.1)	5 (4.9)	0.22 (0.0 to 2.1)^3^	0.219

1Hazard ratio

2Mean difference

3Odds ratio

Variables with a *p* value below 0.1 in the univariate Cox regression analyses (randomization to the restrictive fluid group, a serum lactate at randomization of 2 mmol/L or more, vasopressor use at baseline, a SOFA score of 4 or more at randomization, presence of neoplasia with metastasis, presence of neoplasia without metastasis, ARDS at baseline, and age of 60 years or above) were included in the multivariate analysis. After adjustment, randomization to the restrictive fluid group was independently associated with a lower risk of death before discharge home by day 90 (adjusted HR 0.45, 95% CI 0.24 to 0.83) (Table S3). All included variables showed weak (< 0.4) correlation coefficients (Fig. S4). The VIF of the variables included in the final multivariate Cox regression model was below 2.5, suggesting a low risk of multicollinearity (Table S4).

### Secondary efficacy outcomes

**Fig. 4:**
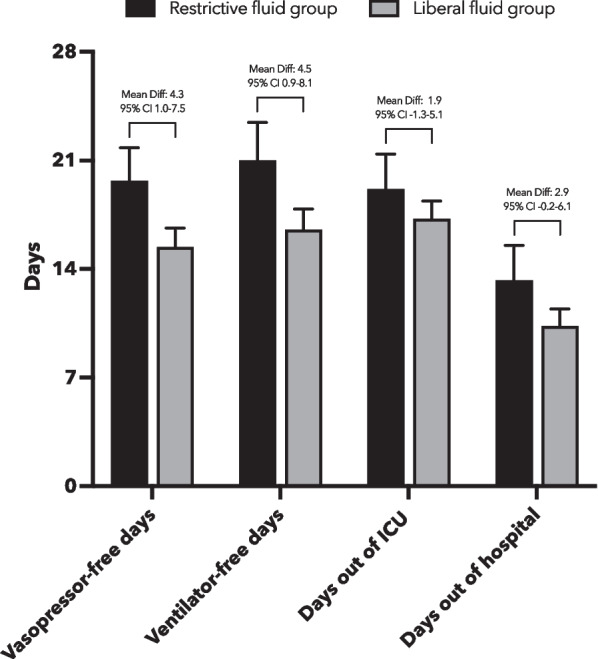
Key secondary outcomes in number of days by day 28 after randomization between the restrictive fluid group and liberal fluid group. Bars with error bars indicate means and 95% confidence intervals

The number of vasopressor-free days by day 28 was significantly lower in the restrictive fluid group (mean ± SD, 19.7 ± 10.4 days) than in the liberal fluid group (mean ± SD, 15.4 ± 12.6 days), with a mean difference of 4.3 days (95% CI 1.0 to 7.5) (Table 2 and Fig. 4). There were significantly more ventilator-free days by day 28 in the restrictive fluid group (21.0 ± 11.8 days) than in the liberal fluid group (16.5 ± 13.6 days), with a mean difference of 4.5 days (95% CI 0.9 to 8.1). The restrictive fluid group had numerically fewer episodes of new onset acute respiratory distress syndrome by day 7 (1 [1.1%] vs. 5 [4.9%]), required fewer intubations by day 28 (12 [14.8%] vs. 18 [19.8%]), experienced more ICU-free days by day 28 (19.1 ± 10.9 vs. 17.2 ± 11.8 days), and had more hospital-free days by day 28 (13.3 ± 11.0 vs. 10.3 ± 11.3 days) than the liberal fluid group, without reaching statistical significance (Table 2 and Fig. 4).

### Kidney function-related outcomes in participants with advanced CKD not previously receiving dialysis

Among participants not previously receiving dialysis, renal-replacement therapy was newly initiated in 6 (12.5%) of 48 participants in the restrictive fluid group and 7 (11.9%) of 59 participants in the liberal fluid group, with a mean number of renal-replacement-free days by day 28 of 20.8 ± 12.0 and 17.1 ± 13.5 days, respectively (mean difference 3.7 days, 95% CI −1.2 to 8.6). In participants with advanced CKD not receiving dialysis, acute kidney injury (stage I, II or III) occurred in 14 (29.2%) of 48 participants in the restrictive fluid group and 11 (18.6%) of 58 participants in the liberal fluid group (OR 1.8, 95% CI 0.7 to 4.9). The occurrence of acute kidney injury stage I (11 [22.9%] vs. 9 [15.3%]), stage II (3 [6.2%] vs. 1 [1.7%]), and stage III (0 [0%] vs. 1 [1.7%]) was similar between the restrictive fluid group and the liberal fluid group (Table S5).

## Discussion

In this *post-hoc* analysis of participants with advanced CKD and sepsis-induced hypotension, we found that a restrictive fluid strategy (prioritizing vasopressor use) was associated with significantly fewer deaths before discharge home by day 90 compared with a liberal fluid strategy. A restrictive fluid strategy was also associated with a greater number of ventilator-free and vasopressor-free days.

The management of sepsis and septic shock remains challenging, with guidance for optimal fluid therapy being largely based on weak recommendations and low-quality evidence [7]. The difficulty of fluid management in sepsis is due to a complex interplay of inflammation-induced endothelial dysfunction, dysregulated osmotic and hydrostatic pressure, and organ failure leading to impaired fluid distribution between the intravascular, interstitial, and intracellular compartments [18]. The recently published SSC Research Priorities 2023 issued a call to investigate a more refined and individualized approach to fluid resuscitation in sepsis and septic shock [19]. Hence, we seized the opportunity to take a closer look at patients with advanced CKD and sepsis-induced hypotension included in the CLOVERS trial and aimed to determine whether a restrictive fluid strategy was beneficial in this patient population.

In our analysis, the benefit of a restrictive fluid protocol was most evident in the subgroup of patients with advanced CKD on dialysis (HR 0.37, 95% CI 0.17 to 0.81). Assignment to the restrictive study arm in participants with advanced CKD excluding participants requiring dialysis was associated with similar but non-significant trend (HR 0.64, 95% CI 0.31 to 1.33). Arguably, failure to achieve statistical significance in the subgroup of participants who had advanced CKD not receiving dialysis (n = 107) may have been due to the limited sample size. However, the 95% confidence interval ranging from 0.31 to 1.33 does not fully exclude potential harm associated with a restrictive fluid approach in patients with advanced CKD not receiving dialysis. The results of this *post-hoc* analysis may lead to the assumption that the greater the chronic impairment of kidney function, the more beneficial a restrictive fluid strategy in sepsis-induced hypotension might be. In contrast, no significant difference between fluid strategies was found in participants without advanced CKD.

Notably, this analysis found that early restrictive fluid strategy within the first 24 h had an impact on the 90 day mortality. This sustained effect might be explained by several physiological effects of fluid overload, which was on average less pronounced the restrictive fluid group (plus 1.1 L) than in the liberal fluid group (plus 2.9 L). Fluid overload may be a particular problem in patients with impaired kidney function due to excess venous volume and interstitial edema, which may explain the beneficial effect of a restrictive fluid strategy in this subgroup. Excessive administration of intravenous crystalloid fluids transiently increases intravascular volume but also leads to worsening extravascular fluid leakage (edema). The latter may interfere with cellular function in the kidneys, liver, heart and lungs [11]. Several days of diuresis after shock resolution are often necessary to remove this excess fluid generated by an initial liberal fluid strategy [20]. Fluid overload of the lung may promote pulmonary edema, requiring prolonged intubation, as it was the case in the liberal fluid group.

The findings of this *post-hoc* analysis emphasize that, in patients with sepsis-induced hypotension, the differentiation between impaired kidney function due to advanced CKD and new-onset sepsis-associated AKI has important clinical implications. Sepsis-associated AKI underlies complex pathophysiological mechanisms, which differ from CKD [20]. While our analysis found a restrictive fluid resuscitation strategy to be beneficial in patients with advanced CKD, another secondary analysis of the CLOVERS trial found no difference between fluid strategies among patients with and without sepsis-associated AKI [21]. Thus, these findings suggest that the clinical context of impaired kidney function (i.e., advanced CKD or sepsis-associated AKI) should be considered in the approach to fluid management of sepsis-induced hypotension.

AKI was numerically more frequent in the restrictive fluid intake group (29%) than in the liberal fluid group (14%), possibly due to reduced renal perfusion. Although the importance of this finding is unclear, the observed reduction in mortality following a restrictive fluid strategy in this analysis outweighs the potential risk of AKI occurrence.

Fluid resuscitation in the initial hours of sepsis onset (as it was done before randomization for all participants in the CLOVERS trial) appears effective across a wide range of patient subgroups with sepsis, including patients with advanced CKD [11]. However, continued large volume infusion of crystalloid fluid might be harmful and confer a risk of volume overload. The results of our analysis might favor a more restrictive fluid approach following initial fluid resuscitation within the first 24 h in patients with sepsis-induced hypotension and advanced CKD.

Our *post-hoc* analysis has limitations. Despite demonstrating a significant trend towards a benefit of a fluid restrictive approach in patients with advanced CKD and sepsis-induced hypotension, this analysis has a limited sample size of only 196 participants and should therefore be regarded as hypothesis-generating as the possibility of type 1 error exists. The sample size of the subgroup of patients not receiving dialysis might have been too small to show any significant effect. Further subgroup analyses were not feasible due to the small sample size. Participants in the restrictive fluid group were slightly younger and had a significantly higher body-mass index [22, 23], which might have affected our results. Mortality in the CLOVERS trial was lower than reported for participants with septic shock in the ICU managed with restrictive or liberal fluid protocols [24], limiting the generalizability of our results. Our analysis did not distinguish between participants receiving continuous hemodialysis or peritoneal dialysis. Although the current or imminent decision to withhold most or all life-sustaining treatment was an exclusion criterion in the COVERS trial, data on treatment withdrawal after inclusion in the trial, potentially influencing the primary outcome, was unavailable.

## Conclusions

In patients with advanced CKD and sepsis-induced hypotension, an early restrictive fluid strategy, prioritizing vasopressor use, was associated with a significantly lower risk of death before discharge home by day 90 as compared with an early liberal fluid strategy. The results of this analysis warrant further clinical investigation to determine whether a restrictive fluid strategy should be favored in patients with advanced CKD and sepsis-induced hypotension.

### Supplementary Information

Additional file1 (PDF 333 kb)

## Data Availability

Access to data from the CLOVERS trial was granted by the NHLBI Biologic Specimen and Data Repository Information Coordinating Center.

## Abbreviations

AKI: Acute kidney injury
ARDS: Acute respiratory distress syndrome
CKD: Chronic kidney disease
CLOVERS: Crystalloid liberal or vasopressor early resuscitation in sepsis
COPD: Chronic obstructive pulmonary disease
ESRD: End-stage renal disease
KDIGO: Kidney disease improving global outcomes
NHLBI: National heart, lung, and blood institute
SOFA: Sequential organ-failure assessment
SSC: Surviving sepsis campaign
