# Supplementary material for: Fluid management for sepsis-induced hypotension in patients with advanced chronic kidney disease: a secondary analysis of the CLOVERS trial
Source: Crit Care. 2024 Jul 11;28:231. doi: 10.1186/s13054-024-05019-6 (PMC11238412; doi:10.1186/s13054-024-05019-6)
Supplement: Supplementary file 1 — Additional file1 (PDF 333 kb) [file 13054_2024_5019_MOESM1_ESM.pdf]

Supplement to:

Fluid Management for Sepsis-Induced Hypotension in Patients with Advanced Chronic Kidney Disease:

A Secondary Analysis of the CLOVERS Trial

Anselm Jorda[1], Ivor S. Douglas[2], Thomas Staudinger[3], Gottfried Heinz[4], Felix Bergmann[1], Rainer Oberbauer[5], Gürkan Sengölge[5], Markus Zeitlinger[1], Bernd Jilma[1], Nathan I. Shapiro[6], Georg Gelbenegger[1]*

[1]Department of Clinical Pharmacology, Medical University of Vienna, Vienna, Austria

[2]Department of Medicine, Pulmonary Sciences and Critical Care, Denver Health and University of Colorado, Anschutz Medical Campus, Denver, CO

[3]Department of Medicine I, Medical University of Vienna, Vienna, Austria

[4]Department of Medicine II, Division of Cardiology, Medical University of Vienna, Vienna, Austria

[5]Department of Medicine III, Division of Nephrology and Dialysis, Medical University of Vienna, Vienna, Austria

[6]Department of Emergency Medicine, Beth Israel Deaconess Medical Center, Harvard Medical School, Boston, MA

Running head: Fluid management in sepsis and advanced CKD

Corresponding Author

Georg Gelbenegger, MD, PhD

Department of Clinical Pharmacology, Medical University of Vienna

Waehringer Guertel 18-20, 1090 Vienna, Austria

georg.gelbenegger@meduniwien.ac.at

Supplementary Tables

Table S1 Baseline characteristics stratified by status of chronic kidney disease (CKD): Advanced CKD not on dialysis, advanced CKD on dialysis, and no advanced CKD.

	Advanced CKD not on dialysis	Advanced CKD on dialysis	No advanced CKD	p
Number of participants	107	89	1367	
Age, years (median (IQR])	69 (57 to 76)	62 (54 to 69)	60 (49 to 70)	<0.001
BMI, kg/m^2 (median [IQR])	27.3 (23.6 to 34.9)	27.8 (23.4 to 36.8)	26.8 (22.5 to 34.0)	0.146
Male sex (%)	53 (49.5)	55 (61.8)	718 (52.5)	0.184
Ethnicity (n [%])				0.347
Hispanic or Latino	21 (19.6)	14 (15.7)	191 (14.0)	
Not Hispanic or Latino	80 (74.8)	70 (78.7)	1124 (82.2)	
Not reported	6 (5.6)	5 (5.6)	52 (3.8)	
Race (n [%])				
Asian	4 (3.7)	4 (4.5)	46 (3.4)	0.841
White	65 (60.7)	45 (50.6)	995 (72.8)	<0.001
African American	22 (20.6)	32 (36.0)	193 (14.1)	<0.001
Not reported	14 (13.1)	9 (10.1)	122 (8.9)	0.347
Chronic obstructive pulmonary disease (%)	21 (19.6)	10 (11.2)	220 (16.2)	0.280
Heart failure (n [%])	20 (18.7)	22 (24.7)	136 (10.0)	<0.001
Hypertension (n [%])	72 (67.3)	54 (60.7)	581 (42.9)	<0.001
Coronary artery disease (n [%])	24 (22.4)	19 (21.3)	173 (12.8)	0.002
Neoplasia (n [%])				0.322
Not present	84 (78.5)	79 (88.8)	1090 (80.5)	
Present	10 (9.3)	3 (3.4)	97 (7.2)	
Present with metastasis	13 (12.1)	7 (7.9)	167 (12.3)	
Diabetes (n [%])				<0.001
Not present	55 (51.4)	46 (51.7)	1003 (74.1)	
Present with end organ damage	21 (19.6)	29 (32.6)	71 (5.2)	
Uncomplicated	31 (29.0)	14 (15.7)	280 (20.7)	
Location of Randomization (n [%])				0.110
Emergency department	96 (89.7)	76 (85.4)	1265 (92.5)	
ICU	11 (10.3)	12 (13.5)	83 (6.1)	
Operating room	0 (0.0)	0 (0.0)	1 (0.1)	
Ward	0 (0.0)	0 (0.0)	13 (1.0)	
Other	0 (0.0)	1 (1.1)	5 (0.4)	
SOFA score at randomization (median [IQR])	5 (3 to 7)	3 (2 to 5)	3 (1 to 5)	<0.001
Volume administered before randomization (mean ± SD)	1886 ± 680	1806 ± 606	1989 ± 624	0.010
Vasopressor use before randomization (n [%])	21 (19.6)	31 (34.8)	252 (18.4)	0.001
Central line	6 (5.6)	10 (11.2)	69 (5.0)	
Peripheral line	12 (11.1)	18 (20.2)	162 (11.9)	
Both	3 (2.8)	3 (3.4)	21 (1.5)	
Vasopressor use at randomization (n [%])	21 (19.6)	29 (32.6)	235 (17.2)	0.001
Acute respiratory distress syndrome at randomization (n [%])	2 (1.9)	2 (2.2)	38 (2.8)	0.822
Serum lactate at randomization, mmol/L (median [IQR))	2.59 (1.58 to 4.10)	2.65 (1.60 to 4.40)	2.10 (1.40 to 3.30)	0.008
Mechanically ventilated at randomization (%)	28 (26.2)	27 (30.3)	286 (21.0)	0.065

Table S2 Therapies Administered during the Trial Intervention Period

	Overall	Restrictive Fluid Group	Liberal Fluid Group	p
Number of participants	196	92	104	
Volume of IV fluid administered over 6 hours, mL (median [IQR])	1325 (352 to 2500)	424 (50 to 950)	2300 (2000 to 3000)	<0.001
Volume of IV fluid administered over 24 hours, mL (median [IQR])	2500 (1050 to 4025)	1200 (490 to 2373)	3325 (2500 to 4641)	<0.001
Urinary output within 24 hours, mL (median [IQR])	380 [0 to 1000]	345 [0 to 989]	390 [0 to 1000]	0.686
Fluid balance over 24 hours, mL (median [IQR])	2115 [750 to 3518]	1087 [90 to 2427]	2961 [1900 to 3995]	<0.001
Vasopressor administration during first 24-hr period (n [%])	120 (61.2)	68 (73.9)	52 (50.0)	0.001
Time from randomization to first vasopressor among participants who had vasopressors administered, hours (mean ± SD)	2.7 ± 4.9	1.6 ± 3.2	4.0 ± 6.3	0.007
Duration of vasopressor use during first 24-hr period among participants who received vasopressor therapy, hours (mean ± SD)	10.5 ± 10.4	13.8 ± 10.2	7.4 ± 9.6	<0.001

Table S3 Univariate and multivariate Cox regression model for death before discharge home by day 90.

	Univariate Analyses				Multivariate Analysis			
Variable	HR	Lower 95% CI	Upper 95% CI	P value	Adj. HR	Lower 95% CI	Upper 95% CI	P value
Serum lactate at randomization ≥2 mmol/L	4.22	1.90	9.36	0.000	3.89	1.72	8.80	0.001
Vasopressor use at baseline	2.45	1.47	4.10	0.001	2.02	1.10	3.70	0.022
SOFA score at randomization ≥4	2.13	1.21	3.78	0.009	1.92	0.99	3.70	0.053
Randomization to restrictive fluid group	0.50	0.29	0.85	0.010	0.45	0.24	0.83	0.011
Neoplasia present with metastasis	2.33	1.21	4.48	0.011	1.86	0.81	4.25	0.142
ARDS at baseline	3.31	1.20	9.15	0.021	2.42	0.72	8.16	0.156
Age ≥60 years	1.95	1.07	3.55	0.028	1.85	0.88	3.90	0.104
Neoplasia present without metastasis	2.24	1.02	4.92	0.045	2.45	1.00	5.96	0.049
COPD	1.54	0.83	2.84	0.169				
Heart failure	1.46	0.83	2.55	0.188				
Chronic dialysis	1.19	0.72	1.97	0.491				
Male Sex	1.20	0.72	2.00	0.495				
Diabetes	0.91	0.55	1.51	0.718				
Hypertension	0.97	0.57	1.64	0.908				

Table S4 Variance inflation factors of the variables included in the final multivariate Cox regression model.

Variable	VIF
Serum lactate at randomization ≥2 mmol/L	1.05
Vasopressor use at baseline	1.10
SOFA score at randomization ≥4	1.14
Randomization to restrictive fluid group	1.10
Neoplasia present with metastasis	1.07
ARDS at baseline	1.08
Age ≥60 years	1.06
Neoplasia present without metastasis	1.08

Table S5 Kidney function-related outcomes.

	Overall	Liberal Fluid Group	Restrictive Fluid Group	P value
N	107	59	48	
Days free from renal-replacement therapy at 28 days (mean (SD))	18.76 (12.94)	17.12 (13.51)	20.77 (12.03)	0.147
Initiation of renal-replacement therapy by 28 days (%)	13 (12.1)	7 (11.9)	6 (12.5)	1
Acute kidney injury at 7 days (%)	25 (23.4)	11 (18.6)	14 (29.2)	0.294
Stage I	20 (18.7)	9 (15.3)	11 (22.9)	0.446
Stage II	4 (3.7)	1 (1.7)	3 (6.2)	0.47
Stage III	1 (0.9)	1 (1.7)	0 (0.0)	1

Supplementary Figures

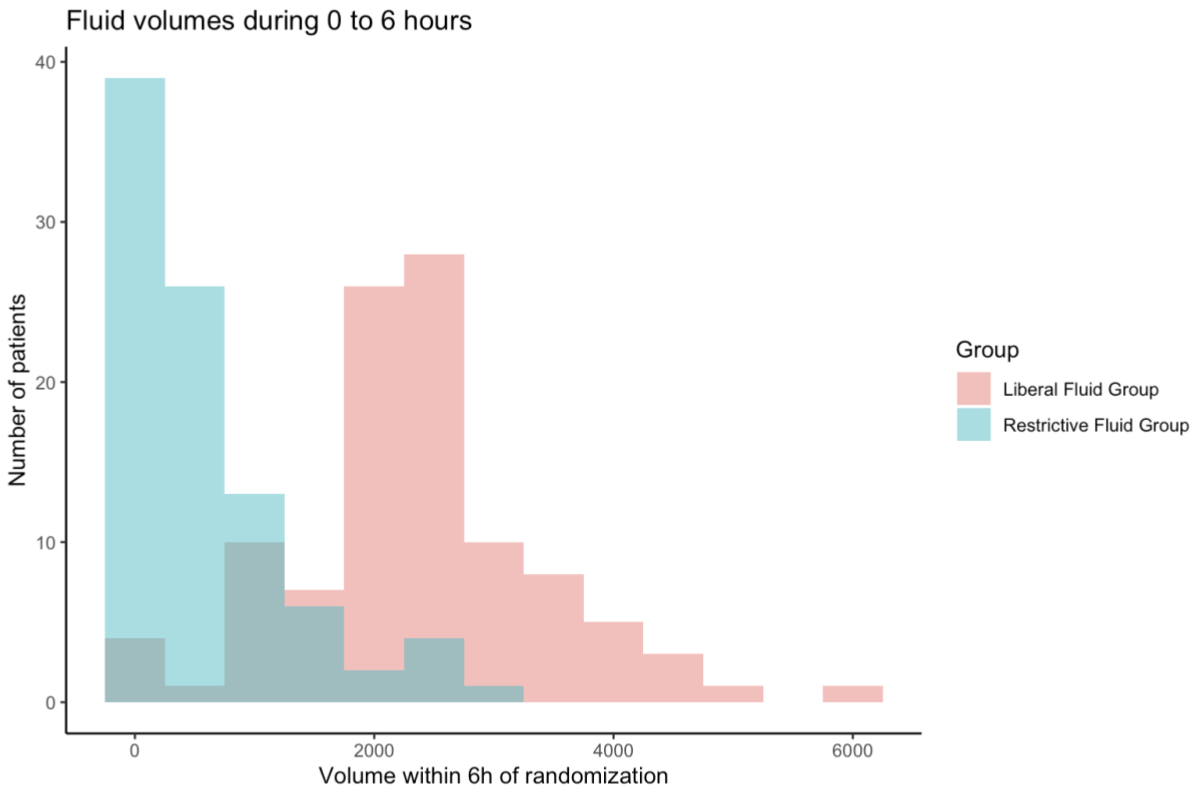

Figure S1 Fluid volume administered within 6 hours from randomization, stratified by treatment groups.

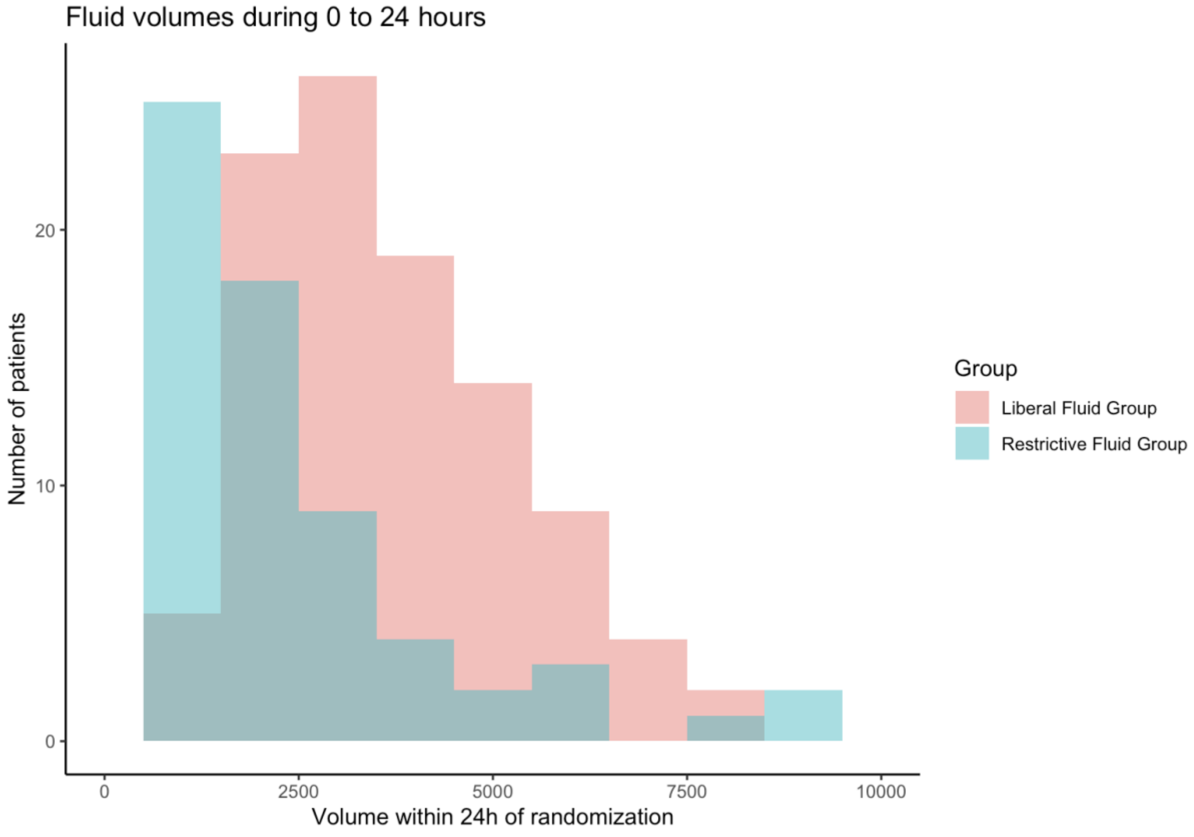

Figure S2 Fluid volume administered within 24 hours from randomization, stratified by treatment groups.

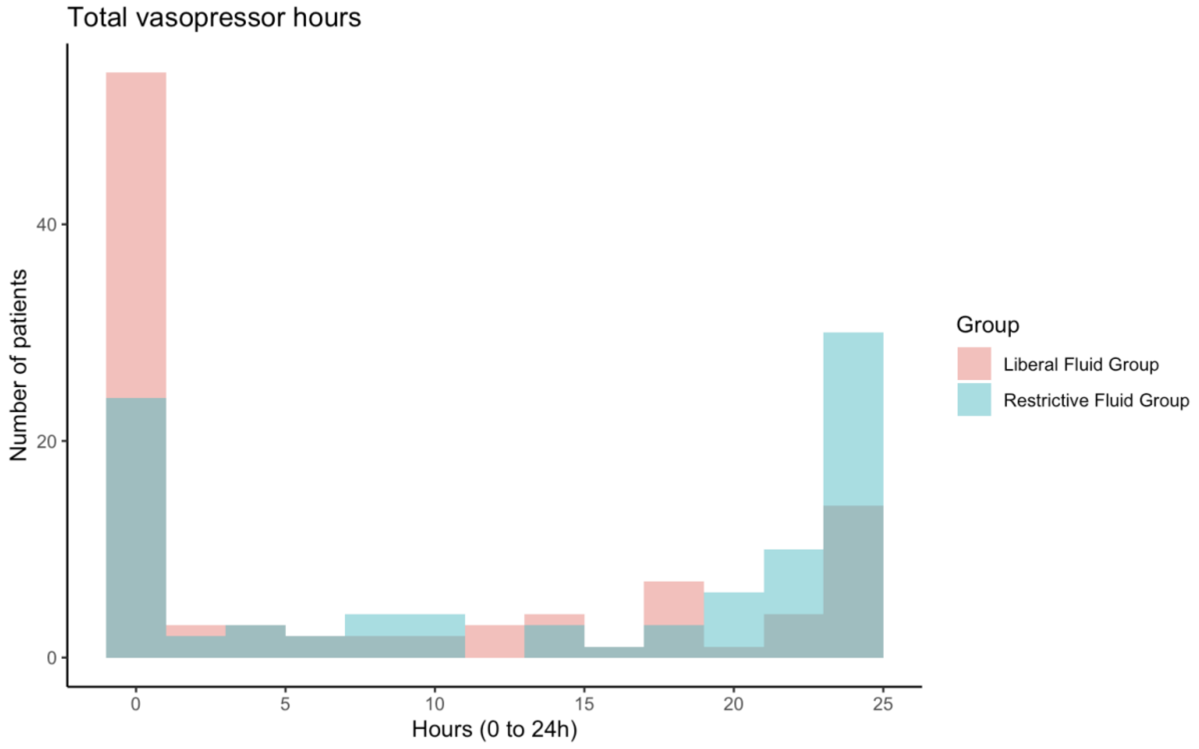

Figure S3 Total vasopressor hours within 24 hours from randomization, stratified by treatment groups.

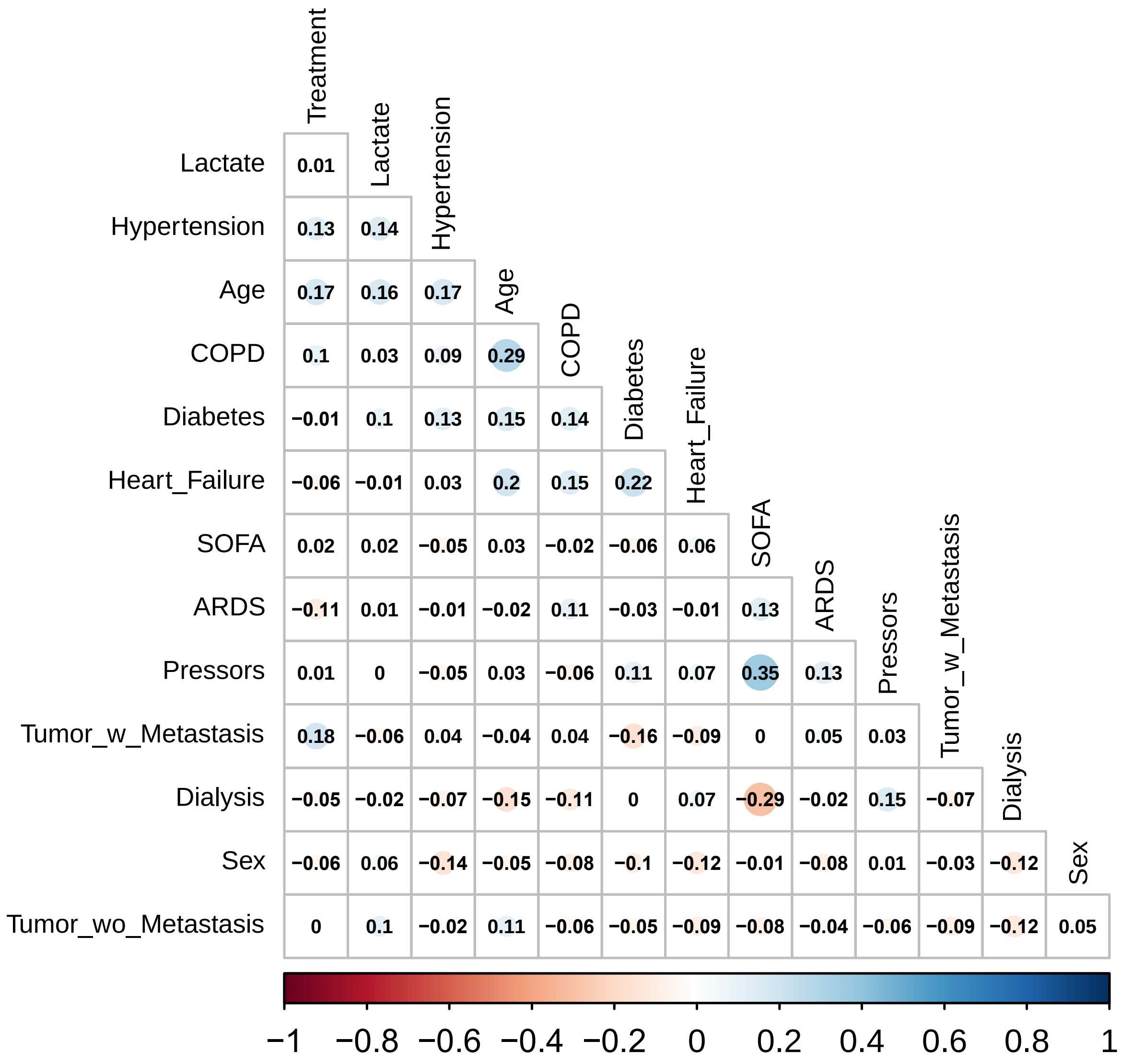

Figure S4 Spearman correlation coefficients between the independent variables.
